# Traumatic right ventricular laceration complicated with hemopericardium, cardiac tamponade, cardiac contusion and epicardial hematoma

**DOI:** 10.1186/2036-7902-7-S1-A33

**Published:** 2015-03-09

**Authors:** Cheng-Hsien Hsieh

**Affiliations:** Emergency department, En-Chu-Kong Hospital, New Taipei City, Taiwan

**Keywords:** Pericardial Effusion, Emergency Physician, Cardiac Tamponade, Blunt Chest Trauma, Chest Injury

## Background

High-energy blunt chest injury will bring out severe cardiac and pulmonary injuries. It may cause cardiac contusion, rupture, cardiac valve dysfunction, and aortic laceration [1]. As traumatic hemopericardium occurs, ultrasound is a convenient and reliable tool for diagnosis. We presented a 55-year-old male victim of blunt chest trauma complicated with hemopericardium, cardiac tamponade, cardiac contusion and epicardial hematoma.

## Case presentation

A 55-year-old drunken male victim presented to the emergency department with chest injury after a car collision accident. The triage showed normal vital sign, and he fell unconscious after standing up. The blood pressure checked immediately was 75/49 mmHg. He regained conscious within 30 seconds and the blood pressure recovered. Focused assessment with sonography for trauma (FAST) performed promptly showed pericardial effusion without fluid around abdominal organs. Parasternal long axis view with curvilinear probe revealed more clearly pericardial effusion, cardiac contusion, and epicardial hematoma. Chest computed tomography was arranged but the patient lost his conscious again with the blood pressure dropped. Repeated FAST got more pericardial effusion and tamponade sign. Pericardiocentesis with 2-way central venous catheter from subxiphoid approach was done smoothly. We drained 60 c.c. bloods out and the blood pressure restored. He was transferred to medical center for cardiovascular surgeon consultation. By the returned medical summary, he was in shock status again there and more than 300 c.c. blood went out from the tube. Emergency median sternotomy was performed and much hemopericardium gushed out. The heart just stopped beating and cardiopulmonary resuscitation began. The bleeder located on the right ventricle next to left anterior descending artery. After right ventricular cardiorrhaphy and successful resuscitation, he was send to intensive care unit with intra-aortic balloon pumping. Total blood lost was about 2000 c.c. His wall ejection fraction was restored in the following echocardiography. He was discharged 13 days later without obvious complication.

## Conclusion

Ultrasound is always a fast and efficient tool for the diagnosis of hemopericardium and intra-abdominal bleeding. Parasternal long axis and short axis views are sometimes more distinct than subxiphoid view of FAST in the field of cardiac injury [2], and may be taken as an alternate. Pericardiocentesis is a life-saving skill that emergency physician should be familiar with, especially when cardiologist is not available. After all, transferring patient to qualified medical center as soon as possible is always the first priority for emergency physicians working in local hospitals.

## Informed consent

The study was conducted in accordance with the ethical standards dictated by applicable law. Informed consent was obtained from each owner to enrolment in the study and to the inclusion in this article of information that could potentially lead to their identification.
